# The shifting burden of gastrointestinal and liver diseases across 11 Southeast Asian nations: a 1990–2021 systematic analysis from the global burden of disease study

**DOI:** 10.2478/abm-2025-0030

**Published:** 2025-10-31

**Authors:** Thanathip Suenghataiphorn, Narisara Tribuddharat, Pojsakorn Danpanichkul, Narathorn Kulthamrongsri

**Affiliations:** 1 Department of Internal Medicine, Griffin Hospital, Derby, CT 06418, USA; 2 Department of Internal Medicine, St. Elizabeth Medical, Boston, MA 02135, USA; 3 Department of Internal Medicine, Texas Tech University Health Science Center, Lubbock, TX 79430, USA; 4 Department of Internal Medicine, University of Hawaii, Honolulu, HI 96822, USA

**Keywords:** gastrointestinal, Global Burden of Disease, liver, regional, Southeast Asia

## Abstract

**Background:**

The burden of gastrointestinal (GI) and liver diseases in Southeast Asian (SEA) nations is substantial and evolving. Understanding contemporary trends is crucial for targeted public health interventions in this heterogeneous region.

**Objective:**

To analyze disability-adjusted life years (DALYs) for major GI and liver diseases across 11 SEA nations from 1990 to 2021.

**Methods:**

Age-standardized DALY rates per 100,000 were extracted from the Global Burden of Disease (GBD) 2021 study. We analyzed overall trends, causes with the highest relative DALY rate increases per country (1990–2021), and countries with the highest DALY rates per cause (2021).

**Results:**

Although infectious GI DALYs such as diarrheal diseases declined, non-communicable disease, particularly GI cancers and chronic liver diseases, showed increasing prominence. Pancreatic cancer DALYs exhibited major relative increases in Indonesia (+61.7%) and Vietnam (+51.6%). Non-alcoholic fatty liver disease (NAFLD)-related DALYs (including cirrhosis) surged in Malaysia (+65.3%) and Thailand (+59.7%). In 2021, Cambodia, Myanmar, and Indonesia faced the highest DALY burdens from chronic hepatitis B and C. Brunei Darussalam, Malaysia, and Thailand led in colorectal cancer DALYs, while Brunei Darussalam also had high pancreatic cancer rates.

**Conclusion:**

SEA nations confront a complex and evolving burden of transitioning GI and liver diseases. The persistent high impact of viral hepatitis, coupled with the alarming rise of NAFLD and GI cancers such as pancreatic cancer, underscores the urgent need for tailored national strategies focused on prevention, early detection, and enhanced treatment access.

Gastrointestinal (GI) and liver diseases impose a significant and escalating global health burden, contributing substantially to morbidity, mortality, and healthcare expenditure [1]. The Southeast Asian (SEA) region, a dynamic area comprising 11 nations with diverse socioeconomic, demographic, and healthcare landscapes, presents a particularly complex and heterogeneous picture of these conditions [2]. Historically, infectious GI ailments and high endemic rates of viral hepatitis have dominated the disease profile in many parts of Southeast Asia [3–5]. However, rapid urbanization, lifestyle modifications, and evolving dietary patterns are driving an epidemiological transition, leading to an increasing prominence of non-communicable GI and liver diseases [6, 7].

Understanding the contemporary trends and inter-country variations in the burden of specific GI and liver diseases across SEA nations is paramount for effective public health planning, resource allocation, and the development of targeted interventions [8, 9]. While regional progress has been made against certain conditions [10, 11], emerging threats such as non-alcoholic fatty liver disease (NAFLD), alcohol-related liver disease [12], and specific GI malignancies are gaining traction, posing new challenges to already strained health systems. Comprehensive, up-to-date analyses using standardized methodologies are crucial to delineate these shifts and identify priority areas for national and regional actions.

The Global Burden of Disease (GBD) study offers a valuable framework for such assessments, providing systematic estimates of disease burden over time [13]. The present study leverages GBD 2021 study data to provide a detailed analysis of the age-standardized disability-adjusted life year (DALY) rates for a comprehensive suite of GI and liver diseases across Brunei Darussalam, Cambodia, Indonesia, Lao People’s Democratic Republic, Malaysia, Myanmar, Philippines, Republic of Singapore, Thailand, Timor-Leste, and the Socialist Republic of Vietnam from 1990 to 2021. Specifically, we aimed to: (1) quantify overall trends in GI and liver disease DALYs; (2) identify the leading GI and liver disease causes within each nation in 2021; (3) determine the top 3 causes exhibiting the highest relative percentage DALY rate increase (or smallest decrease) within each country between 1990 and 2021; and (4) identify the top 3 countries with the highest DALY rates for each GI and liver disease in 2021, thereby mapping the current burden and key evolving challenges in the region.

## Methods

### Data source and study period

The present study utilized publicly available, aggregated data from the GBD 2021 study, coordinated by the Institute for Health Metrics and Evaluation (IHME) [13]. The GBD 2021 study provides a systematic and comprehensive assessment of mortality, morbidity, and risk factors for 371 diseases and injuries across 204 countries and territories, including subnational estimates for some locations. Data were extracted for the period spanning from 1990 to 2021, inclusive, to analyze trends. The GBD study does not conduct primary data collection but synthesizes and models data from multiple sources, including vital registration systems, censuses, household surveys, disease registries, and published scientific literature. The availability and quality of these input data vary significantly across countries and over the 32-year study period. For the 11 SEA nations analyzed, IHME employs standardized modeling techniques to generate complete and comparable estimates, accounting for data gaps and inconsistencies. This process ensures that, although underlying data quality may differ, the final GBD estimates are generated using a consistent and systematic methodology across all locations.

### Study region and disease scope

The analysis focused on 11 SEA nations: Brunei Darussalam, Cambodia, Indonesia, Lao People’s Democratic Republic, Malaysia, Myanmar, Philippines, Republic of Singapore, Thailand, Timor-Leste, and the Socialist Republic of Vietnam. The GBD cause hierarchy is structured into mutually exclusive and collectively exhaustive levels. Level 2 causes represent broad disease or injury groups, such as “Digestive Diseases.” Level 3 causes are more specific diseases within that group (e.g., “Cirrhosis and other chronic liver diseases,” “Colon and rectum cancer”). Level 4 causes provide further etiological detail (e.g., “Liver cancer due to hepatitis B”). Our analysis included the parent Level 2 causes and all their constituent Levels 3 and 4 sub-causes to ensure a comprehensive assessment. We included all causes and categorized them under “Digestive Diseases” (GBD Level 2 cause) and their constituent Level 3 and Level 4 specific diseases as defined in the GBD 2021 cause hierarchy. This encompassed a comprehensive range of GI and liver conditions. Disease definitions and classifications were based on the International Classification of Diseases (ICD) codes mapped within the GBD framework.

### Metric of disease burden

The primary metric analyzed was the age-standardized DALY rate per 100,000 population. DALYs are a summary measure of population health, representing the sum of years of life lost (YLLs) due to premature mortality and years lived with disability (YLDs) [14]. Age-standardization was performed by GBD using its global standard population to facilitate valid comparisons of disease burden across different countries and over time by accounting for variations in population age structures. All estimates are presented with their corresponding 95% uncertainty intervals (UIs), which reflect the statistical uncertainty in the data inputs and modeling process.

### Data analysis and presentation

All analyses were conducted using data for both sexes combined. The age-standardized DALY rates for all included GI and liver diseases combined were calculated for each country by summing the DALY rates of individual GI and liver causes. Trends from 1990 to 2021 were plotted for all 11 SEA nations. For each of the 11 SEA countries, the top 5 specific GI and liver disease causes (based on GBD Level 3 or 4 causes) were identified based on their age-standardized DALY rates in 2021. Trends in DALY rates for these top 5 causes, along with all other GI/liver causes (aggregated as “others”), were plotted from 1990 to 2021. For each country, the percentage change in age-standardized DALY rates between 1990 and 2021 was calculated for all GI and liver causes with available data for both time points and a non-zero DALY rate in 1990. The 3 causes with the highest relative percentage change (most positive or least negative, indicating the greatest relative increase or smallest relative decrease) were identified and presented along with their DALY rates in 1990 and 2021 (with 95% UIs) and the calculated percentage change. For each specific GI and liver disease, the 3 countries with the highest age-standardized DALY rates in 2021 were identified. These countries were presented along with their DALY rates in 2021 (with 95% UIs), their DALY rates in 1990, and the percentage change between 1990 and 2021.

Data processing, analysis, and visualization were performed using R software (version 4.4.2; R Foundation for Statistical Computing) and the tidyverse and gt packages (R Software, Journal of Open Source Software).

### Ethical considerations

The present study utilized exclusively publicly available, aggregated, and de-identified data from the GBD study. No individual patient data were accessed or analyzed. Therefore, specific institutional ethical review board approval was not required for the present study.

## Results

### Overall burden and trends of GI and liver diseases in SEA nations

**Figure 1.**
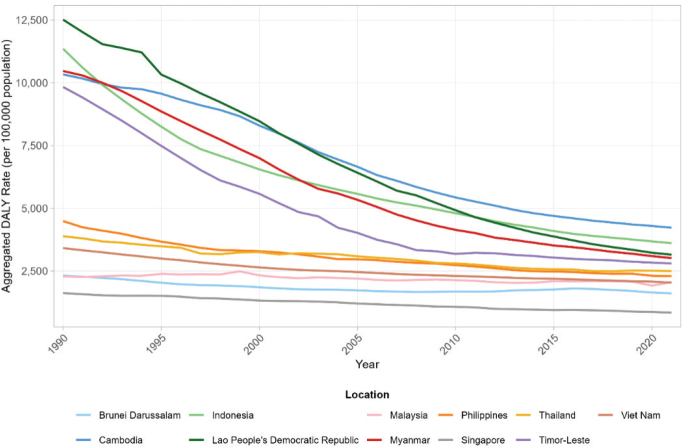
Overall age-standardized DALY rates for GI and liver diseases in SEA nations from 1990 to 2021. DALY, disability-adjusted life year; GI, gastrointestinal; SEA, Southeast Asian.

Substantial variation in the overall age-standardized DALY rates for combined GI and liver diseases was observed across the 11 SEA nations throughout the 1990–2021 study period (Figure 1). In 1990, the Lao People’s Democratic Republic (e.g., ~12,500 DALYs per 100,000) and Myanmar (e.g., ~11,000 DALYs per 100,000) exhibited the highest aggregated burdens, while Brunei Darussalam (e.g., ~2,300 DALYs per 100,000) and Malaysia (e.g., ~2,400 DALYs per 100,000) had the lowest. Most countries demonstrated a decline in this overall burden by 2021, though the extent varied. For instance, the Lao People’s Democratic Republic saw a significant reduction to approximately 4,500 DALYs per 100,000, while Brunei Darussalam’s rate decreased to around 1,700 DALYs per 100,000 by 2021. Despite these declines, considerable DALY rates persisted in several nations.

### Leading GI and liver diseases within countries

The top GI and liver diseases with the highest relative DALY rate increases (1990–2021) varied distinctly (Table 1). Pancreatic cancer DALYs showed significant relative increases in multiple nations, including Indonesia (+61.7%), Vietnam (+51.6%), the Philippines (+46.0%), and Cambodia (+31.0%). Metabolic liver diseases also increased; NAFLD-related DALYs, encompassing NAFLD including cirrhosis and liver cancer due to non-alcoholic steatohepatitis (NASH) increased substantially in Malaysia (e.g., +65.3% for NAFLD with cirrhosis; +62.3% for total NAFLD burden) and Thailand (+59.7% for NAFLD with cirrhosis). Thailand also saw a notable relative rise in peptic ulcer disease DALYs (+271.2%). Other prominent relative increases included colon and rectum cancers in Vietnam (+43.6%) and the Philippines (+30.1%), and liver cancer due to alcohol use in Vietnam (+35.8%) and Cambodia (+23.6%).

**Table 1.** Top 3 GI and liver disease causes with the highest relative percentage change in age-standardized DALY rates by SEA nations from 1990 to 2021

DALY rates per 100,000 population			
Cause of DALYs	DALY rate 1990	DALY rate 2021 (95% UI)	% change (1990–2021)
**Brunei Darussalam**			
Other digestive diseases	31.9	32.8 (27.5–38.7)	+2.9
Pancreatic cancer	129.3	130.4 (106.0–156.9)	+0.8
Gastroesophageal reflux disease	48.6	48.6 (24.5–87.3)	–0.1
**Cambodia**			
Pancreatic cancer	64.3	84.2 (63.4–108.4)	+31.0
Liver cancer due to alcohol use	34.4	42.6 (19.9–87.0)	+23.6
Colon and rectum cancer	333.5	353.1 (262.5–456.9)	+5.9
**Indonesia**			
Pancreatic cancer	58.9	95.3 (68.8–122.1)	+61.7
Liver cancer due to NASH	8.5	11.9 (6.4–18.4)	+38.8
Colon and rectum cancer	239.1	297.6 (223.3–371.8)	+24.5
**Lao People’s Democratic Republic**			
Pancreatic cancer	64.6	75.6 (54.3–102.6)	+17.0
Gastroesophageal reflux disease	41.2	41.2 (20.9–74.3)	+0.1
Gallbladder and biliary diseases	112.1	108.4 (76.3–146.2)	–3.4
**Malaysia**			
NAFLD including cirrhosis	11.8	19.5 (12.4–28.1)	+65.3
Total burden related to NAFLD	21.6	35.1 (26.4–46.9)	+62.3
Liver cancer due to NASH	9.9	15.7 (10.3–22.5)	+58.8
**Myanmar**			
Pancreatic cancer	62.8	75.7 (55.6–100.2)	+20.5
Liver cancer due to alcohol use	12.7	14.3 (7.0–31.8)	+12.4
Liver cancer due to NASH	7.3	7.4 (3.2–13.7)	+ 1.3
**Philippines**			
Pancreatic cancer	60.5	88.4 (73.4–105.6)	+46.0
Colon and rectum cancer	263.2	342.3 (287.2–402.3)	+30.1
NAFLD including cirrhosis	11.9	13.0 (8.9–18.1)	+9.9
**Singapore**			
Paratyphoid fever	0.0	0.0 (0.0–0.0)	+14.1
Gastroesophageal reflux disease	51.5	51.8 (25.7–94.0)	+0.7
Pancreatic cancer	123.7	106.6 (98.3–114.4)	–13.8
**Thailand**			
Peptic ulcer disease	8.2	30.4 (24.1–38.7)	+271.2
NAFLD including cirrhosis	21.8	34.8 (21.9–52.4)	+59.7
Gastritis and duodenitis	20.2	26.2 (18.2–36.7)	+30.0
**Timor-Leste**			
Pancreatic cancer	41.1	53.2 (41.0–67.7)	+29.4
Colon and rectum cancer	212.8	227.2 (171.7–296.9)	+6.8
Gastroesophageal reflux disease	40.9	41.0 (20.8–73.8)	+0.3
**Viet Nam**			
Pancreatic cancer	35.2	53.4 (38.9–66.4)	+51.6
Colon and rectum cancer	208.8	299.9 (228.8–365.5)	+43.6
Liver cancer due to alcohol use	69.7	94.7 (57.2–150.6)	+35.8

1 Only causes with data for both 1990 and 2021 and a non-zero 1990 DALY rate are included in the percent change calculation.

1 DALY, disability-adjusted life year; NAFLD, non-alcoholic fatty liver disease; NASH, non-alcoholic steatohepatitis; UI, uncertainty interval.

In 2021, the distribution of high-burden countries differed by specific GI and liver diseases (Table 2). Cambodia, Myanmar, and Indonesia consistently ranked among the top 3 for DALYs from chronic hepatitis B and C, including cirrhosis, and cirrhosis due to alcohol. Despite major declines, diarrheal diseases remained most burdensome in Indonesia (941.7 DALYs per 100,000), the Lao People’s Democratic Republic (736.5), and Timor-Leste (721.7). Higher-income nations like Brunei Darussalam, Malaysia, and Thailand exhibited the highest DALY rates for colon and rectum cancers, while Brunei Darussalam also led for pancreatic cancer. Liver cancer due to NASH was most prominent in Thailand (27.7 DALYs per 100,000), Vietnam (25.9), and Malaysia (15.7).

**Table 2.** Top 3 SEA nations by age-standardized DALY rates for each selected GI and liver disease, 2021

DALY rates per 100,000 population. Percent change relative to 1990			
Country	DALY rate 2021 (95% UI)	DALY rate 1990	% change (1990–2021)
**Appendicitis**			
Cambodia	40.5 (24.7–66.7)	103.3	–60.8
Myanmar	30.9 (19.9–48.2)	101.4	–69.5
Timor-Leste	27.0 (16.0–55.9)	55.5	–51.4
**Chronic hepatitis B including cirrhosis**			
Cambodia	563.3 (371.6–781.8)	1,053.0	–46.5
Myanmar	338.7 (216.8–470.6)	618.2	–45.2
Indonesia	327.8 (264.8–420.8)	531.1	–38.3
**Chronic hepatitis C including cirrhosis**			
Cambodia	470.1 (305.2–670.1)	818.0	–42.5
Indonesia	468.1 (374.9–581.9)	708.2	–33.9
Myanmar	262.0 (170.3–372.4)	465.2	–43.7
**Cirrhosis due to alcohol**			
Cambodia	351.2 (225.6–523.6)	481.4	–27.0
Indonesia	190.4 (146.0–245.7)	266.9	–28.7
Myanmar	184.3 (110.9–276.0)	283.3	–34.9
**Colon and rectum cancers**			
Brunei Darussalam	466.4 (393.7–547.7)	674.4	–30.8
Malaysia	418.7 (364.6–469.1)	399.4	+4.8
Thailand	380.3 (287.9–486.3)	320.2	+ 18.8
**Diarrheal diseases**			
Indonesia	941.7 (649.5–1190.9)	7454.6	–87.4
Lao People’s Democratic Republic	736.5 (511.3–1047.9)	8292.4	–91.1
Timor-Leste	721.7 (498.5–1011.0)	7015.5	–89.7
**Gallbladder and biliary tract cancers**			
Thailand	150.3 (74.0–207.8)	156.3	–3.9
Brunei Darussalam	53.4 (41.1–68.1)	86.2	–38.1
Viet Nam	27.2 (18.4–40.8)	25.1	+8.5
**Gastroesophageal reflux disease**			
Singapore	51.8 (25.7–94.0)	51.5	+0.7
Brunei Darussalam	48.6 (24.5–87.3)	48.6	–0.1
Indonesia	43.4 (21.7–78.1)	43.4	+0.0
**Inflammatory bowel disease**			
Brunei Darussalam	16.3 (12.8–21.7)	20.1	–18.8
Cambodia	10.3 (6.3–14.6)	13.7	–25.0
Indonesia	8.9 (6.3–10.8)	12.9	–31.1
**Liver cancer due to NASH**			
Thailand	27.7 (17.7–42.3)	31.1	–11.0
Viet Nam	25.9 (16.5–39.0)	26.6	–2.6
Malaysia	15.7 (10.3–22.5)	9.9	+58.8
**Liver cancer due to alcohol use**			
Viet Nam	94.7 (57.2–150.6)	69.7	+35.8
Thailand	87.7 (58.3–126.9)	105.2	–16.7
Lao People’s Democratic Republic	47.8 (28.0–72.0)	59.9	–20.2
**Liver cancer due to hepatitis B**			
Viet Nam	161.3 (106.4–246.1)	225.0	–28.3
Thailand	133.1 (91.9–186.6)	202.9	–34.4
Brunei Darussalam	118.3 (84.0–161.1)	202.5	–41.6
**Liver cancer due to hepatitis C**			
Brunei Darussalam	79.3 (52.1–110.6)	112.4	–29.5
Viet Nam	50.8 (29.5–80.6)	61.8	–17.8
Cambodia	44.5 (23.5–78.4)	61.8	–28.0
**Pancreatic cancer**			
Brunei Darussalam	130.4 (106.0–156.9)	129.3	+0.8
Thailand	118.8 (91.6–154.1)	91.9	+29.3
Singapore	106.6 (98.3–114.4)	123.7	–13.8
**Pancreatitis**			
Cambodia	68.1 (42.7–107.9)	80.9	–15.8
Myanmar	63.0 (37.0–119.0)	80.3	–21.5
Malaysia	59.8 (46.2–80.0)	63.9	–6.5
**Peptic ulcer disease**			
Cambodia	552.2 (327.1–868.7)	1175.8	–53.0
Timor-Leste	342.4 (227.4–522.8)	556.0	–38.4
Lao People’s Democratic Republic	327.3 (232.0–457.9)	833.0	–60.7
**Total burden related to NAFLD**			
Thailand	62.5 (43.0–87.2)	52.9	+18.1
Indonesia	58.0 (40.7–79.0)	48.5	+19.7
Cambodia	53.2 (34.9–80.5)	68.0	–21.8

1 N/A indicates data not available or cause not in the top 3 for any country.

1 DALY, disability-adjusted life year; GI, gastrointestinal; NAFLD, Non-alcoholic fatty liver disease; UI, uncertainty interval.

### Country-specific leading causes and trends

**Figure 2.**
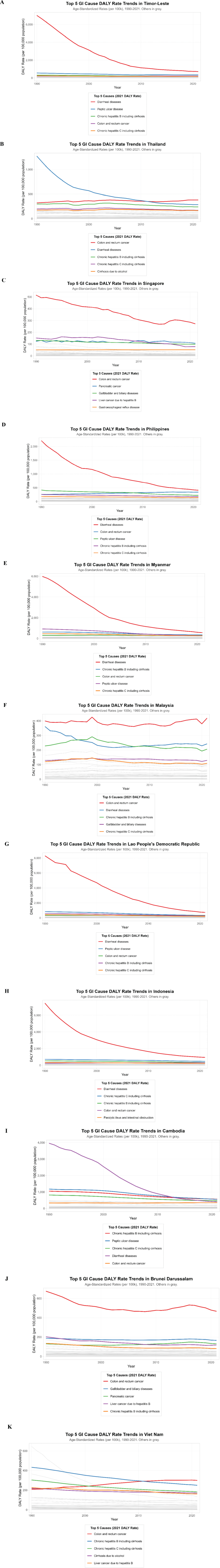
(A) Top 5 GI cause DALY rate trends in Timor-Leste, (B) Thailand, (C) Singapore, (D) Philippines, (E) Myanmar, (F) Malaysia, (G) Laos, (H) Indonesia, (I) Cambodia, (J) Brunei Darussalam, (K) Vietnam. DALY, disability-adjusted life year; GI, gastrointestinal.

The 5 leading specific causes of GI and liver DALYs in 2021 for each of the 11 SEA nations, and their trends since 1990, are detailed in Figures 2A–K. These plots illustrate diverse epidemiological profiles. Overall, the findings illustrate a distinct epidemiological transition across SEA. Significant reductions in the burden of infectious conditions such as diarrheal diseases were evident in most countries between 1990 and 2021. Concurrently, non-communicable diseases (NCDs) including various GI cancers (colon/rectum, stomach) and chronic liver diseases (due to viral hepatitis, alcohol, and potentially metabolic factors), constituted a large and, in some cases, relatively increasing proportion of the GI and liver disease burden, particularly in higher-income and transitioning nations. However, substantial burdens from both infectious and non-communicable GI and liver conditions persisted across the region in 2021.

## Discussion

The present study, utilizing comprehensive GBD 2021 study data from 1990 to 2021, reveals the substantial and dynamic burden of GI and liver diseases across 11 SEA nations. The principal findings of the present study underscore 3 key themes: (1) the sheer scale and persistent nature of the digestive disease burden in the region, (2) the profound heterogeneity in disease patterns and trends among these countries, and (3) the clear evidence of an ongoing, yet incomplete, epidemiological transition, with varying balances between infectious and non-communicable causes.

The overarching decline in total GI and liver DALYs observed in most SEA nations likely reflects broad public health improvements, including enhanced sanitation, access to cleaner water, and improved primary healthcare, which have notably curtailed the impact of historically dominant infectious conditions such as diarrheal diseases [15]. However, the considerable variation in current DALY rates and the magnitude of decline between countries—with nations such as the Lao People’s Democratic Republic and Myanmar still facing a much higher overall burden compared with Singapore or Brunei—highlights persistent disparities in health system capacity and socioeconomic development [16, 17]. The progress is therefore uneven, reflecting deep-seated differences in health system capacity and infrastructure across the region.

We found that while infectious diseases declined, NCDs became increasingly prominent. This epidemiological shift, however, must be interpreted within the context of the region’s rapid socioeconomic and healthcare evolution. For instance, the concerning relative DALY rate increases for pancreatic cancer across 7 SEA nations, including Indonesia (+61.7%) and Vietnam (+51.6%), may reflect not only a true rise in incidence due to risk factors such as smoking, obesity, and diabetes but also improved diagnostic capabilities. Over the past 3 decades, the wider availability of advanced imaging technologies (e.g., CT, MRI) in urban centers would naturally lead to increased detection of previously occult malignancies, thereby inflating DALY rates. Similarly, the surge in NAFLD-related DALYs, particularly in economically transitioning nations such as Malaysia (+65.3%) and Thailand (+59.7%), points to a growing metabolic health crisis driven by changing diets and sedentary lifestyles [18, 19]. However, this trend is also amplified by greater clinical awareness and screening practices. Specific disease hotspots in 2021 reveal persistent challenges. High diarrheal disease rates continue in Indonesia, Lao People’s Democratic Republic, and Timor-Leste, likely reflecting ongoing deficiencies in water, sanitation, and hygiene (WASH) infrastructure that require sustained public health investment [20]. Cambodia, Myanmar, and Indonesia show concentrated burdens of chronic hepatitis B and C, emphasizing the need for robust viral hepatitis elimination programs that may face hurdles related to public health funding, screening coverage, and linkage to care [21]. In stark contrast, the prominence of NCDs such as colorectal cancer (Brunei, Malaysia, and Thailand) and pancreatic cancer (Brunei, Thailand, and Singapore) in higher-income nations underscores the dual influence of lifestyle-related risk factors and more established healthcare systems capable of diagnosing these conditions more effectively [22]. These divergent patterns, visualized in supplementary country plots, underscore the complex epidemiological transition across SEA.

These findings underscore profound public health implications for SEA nations. The region’s heterogeneity demands tailored national strategies, moving beyond a “one-size-fits-all” approach, informed by country-specific DALY profiles, socioeconomic context, and health system readiness, akin to a form of public health precision medicine [23]. While continued efforts against infectious diseases are vital in high-burden areas, a strategic regional pivot toward preventing and managing NCDs—particularly GI cancers, NAFLD, and alcohol-related liver disease—is crucial. This necessitates integrated primary (lifestyle, alcohol control, and HBV vaccination) [24, 25], secondary (cancer/hepatitis screening) [26], and tertiary (treatment access) prevention [27]. The alarming rise in pancreatic cancer and the burgeoning NAFLD epidemic require dedicated research into drivers and proactive public health responses, including awareness and primary care integration. Future research should prioritize subnational burden analyses to guide targeted interventions in larger nations and investigate determinants of increasing NCDs such as pancreatic cancer and NAFLD. Cost-effectiveness studies for screening and treatment programs within diverse SEA settings are also essential.

The strengths of the present study lie in its comprehensive use of GBD 2021 study data, providing standardized, comparable estimates across 11 countries for more than 32 years. This allows for a robust analysis of long-term trends and regional patterns. However, the findings must be interpreted in light of several important limitations. First, the study is subject to the inherent limitations of the GBD methodology itself. GBD estimates are the output of complex statistical models, and their precision is contingent on the quantity and quality of the primary input data, which varies substantially by country, disease, and year. For some nations and causes in this analysis, estimates may be based on sparse data, leading to wider UIs and less robust conclusions. Second, as an ecological study, our findings demonstrate associations at the population level and cannot be used to infer individual-level risk—the ecological fallacy. Third, this analysis of national-level data may mask significant subnational disparities in disease burden, particularly within large and diverse archipelagic nations such as Indonesia and the Philippines. Finally, changes in GBD modeling methodology or disease classification systems (based on ICD updates) over time could potentially influence some of the observed trends, independent of true epidemiological shifts.

## Conclusion

In conclusion, SEA nations confront a complex and evolving burden of GI and liver diseases, characterized by a dual challenge. While successes against infectious diseases are evident, a formidable and growing threat from NCDs—including specific GI cancers and metabolic liver disease—now requires an urgent strategic pivot. The profound heterogeneity observed means that a “one-size-fits-all” approach is inadequate. Instead, national health policies must be precisely tailored, informed by country-specific DALY profiles and their underlying drivers, including socioeconomic status and healthcare capacity. This requires a dual-pronged public health response: continuing to strengthen WASH infrastructure and viral hepatitis programs in high-burden areas, while simultaneously implementing robust strategies for NCDs focused on primary prevention (e.g., metabolic risk factor control), targeted secondary prevention (e.g., cancer screening), and equitable access to advanced diagnostics and treatment.
